# Optical Properties of Ag Nanoparticle Arrays: Near-Field Enhancement and Photo-Thermal Temperature Distribution

**DOI:** 10.3390/nano12213924

**Published:** 2022-11-07

**Authors:** Daobin Luo, Pengcheng Hong, Chao Wu, Shengbo Wu, Xiaojing Liu

**Affiliations:** 1 School of Arts and Sciences, Shaanxi University of Science & Technology, Xi’an 710021, China; 2 Xian Institute of Space Radio Technology, Xi'an 710000, China

**Keywords:** Ag nanoparticles, square arrays, near-field enhancement, photo-induced heating, photothermal temperature distribution

## Abstract

The near-field and photo-thermal properties of nanostructures have always been the focus of attention due to their wide applications in nanomaterials. In this work, we numerically investigate the near-field and photo-thermal temperature distribution in a nanoparticle array when the scattering light field among particles is considered. ‘Hot spots’, which represent strong electric field enhancement, were analyzed at the difference of the particle size, particle spacing and the polarization direction of the incident light. Interestingly, it is found that the position of the ‘hot spots’ does not rotate with the polarization direction of the incident light and always remains in the particle gaps along the line between particle centers. Moreover, the near-field is independent of the polarization in some special areas, and the factor of near-field enhancement keeps constant in these spots when the illumination polarization varies. As for photo-induced heating, our results show that both the temperature of the structure center and maximum temperature increase linearly with the particle number of the array while decreasing with the increase in particle spacing. This work provides some theoretical considerations for the near-field manipulation and photo-thermal applications of nanoarrays.

## 1. Introduction

Noble metal nanoparticles have attracted extensive interest in physics, chemistry, and biology due to their surface plasmon properties. When the free charges oscillate, collectively driven by the incident light at resonance frequency, the local surface plasmon (LSP) phenomenon occurs [1]. Noble metal nanoparticles have optical properties of strong light absorption and near-field enhancement in the visible band when LSP occurs. The LSP of noble metal nanoparticles has been widely studied in the last 20 years [2]. Researchers have made great contributions to the optical properties of nanoparticles with different shapes [3] and of periodic array structures [4,5]. Our group has also studied the factor of near-field enhancement and determined the near-field distribution of Ag dimer [6]. The optical properties of nanoparticles have been widely applied in surface enhanced Raman scattering [7], photovoltaic [8,9], photocatalytic and other fields [10].

At the same time, the strong light absorption of noble metal nanoparticles in the visible light band increases the temperature of the nanoparticles themselves, and the photo-thermal effect is another important property of plasmonic structures [11]. For a long time in the past, the photo-thermal effect was regarded as a negative effect by researchers and was reduced or suppressed [12]. Later, researchers gradually found that the photo-thermal properties of nanoparticles could be applied to thermos-physics [13], biomedicine and other fields [14], and it has attracted increasing attention since [15]. Now, the photo-thermal effect of nanostructure has been widely used in the fields of photo-thermal therapy [16], drug release [17], solar energy collection [18] and other areas.

Due to the long-range ordered periodicity, nanoarrays have the characteristics of expanding the electromagnetic enhancement space, generating collective lattice resonances [4,5], increasing scattering efficiency, and enhancing thermal accumulation effects [19]. Thus, we investigated the absorption, scattering, near-field enhancement and photothermal properties of Ag nanoarrays. In this article, discrete dipole approximation (DDA) and thermal Green's function method are introduced to investigate near-field enhancement and photo-thermal temperature distribution, respectively. The influences of particle radius, particle spacing, particle number of the array and illumination polarization angle on the unique optical properties of Ag nanoarrays are discussed. The purpose of this work is to understand the near-field and photo-thermal temperature distribution of Ag nanoarrays and to explore a method for designing the nanostructure in photo-thermal and near-field regulation.

## 2. Theories and Methods

**Figure 1:**
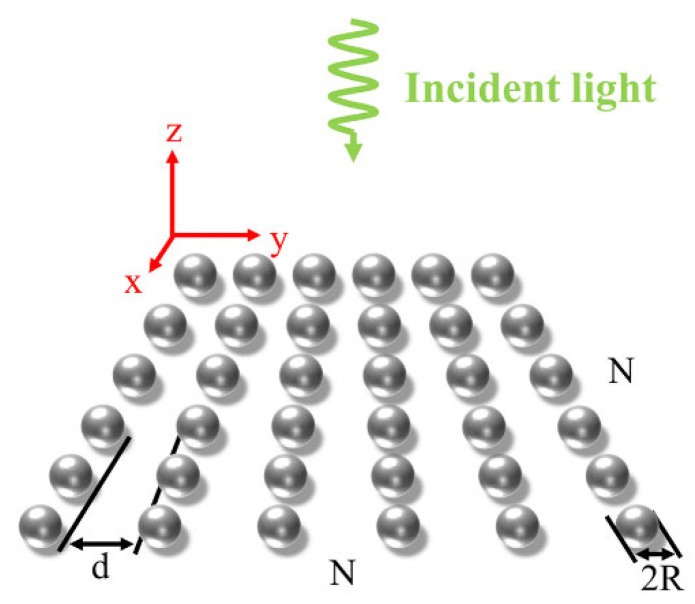
Schematic diagram of square array of spherical nanoparticles.

Figure 1 is a schematic diagram of a square array of spherical nanoparticles. R and d denote the particle radius and spacing, respectively. The plane of the array is located at the xoy and the center of the array structure is located at the coordinate origin. The incident light travels along the negative direction of the z-axis.

We used DDA to study the near-field enhancement, absorption, and scattering properties of the nanoparticle array. Based on the absorption properties of nanoparticles and thermal Green's function method, the photo-thermal temperature distribution was investigated.

### 2.1. Discrete Dipole Approximation Method

DDA is a method used to calculate the scattering and absorption of electromagnetic wave radiation by targets with an arbitrary geometry whose size is less than or equal to the wavelength of incident light [20]. The main idea is to treat the target model as a set of multiple point dipoles. Each point dipole occupies a position in the target lattice. They are polarized to generate an electric dipole moment when the incident electromagnetic wave illuminates the target. Then, each point dipole is affected not only by the external electric field but the scattering field of other surrounding point dipoles. Therefore, the electromagnetic field around the target point dipole is the superposition of the incident field and the scattering field of the other multiple point dipoles. Draine and Flatau [21] developed a ddscat7.3 program code based on DDA theory to calculate the electric field distribution of electromagnetic scattering from various nanostructures. In this article, ddscat7.3 is used to calculate the absorption, scattering, and near-field enhancement of the Ag nanoparticle array.

### 2.2. Thermal Green's Function Method

As for the plasma thermal behavior of metal nanoparticle assembly, we commonly use the thermoplasmonics theory [15]. The thermal effect produced by the interaction between incident light and particles increases the temperature of the particles themselves and the temperature of the surrounding medium increases due to thermal diffusion. As shown in Figure 1, the heat absorption power of the ith nanoparticle in the structure is:

(1) $$Q_{i}=\frac{1}{2}nc\varepsilon_{0}\sigma_{abs}{\left|E_{i}^{ext}\right|}^{2},$$

where n stands the refractive index of the surrounding medium, c stands the speed of light, $\varepsilon_{0}$ stands the dielectric constant of vacuum, $E_{i}^{ext}$ stands the external electric field intensity around the particles after the incident light interacts with the nanoparticle array. The electric field intensity can be calculated using the DDA method. Siahpoush et al. [22] used this method in the study of the effect of plasmonic coupling on the photo-thermal behavior of random nanoparticles. $\sigma_{abs}$ stands the absorption cross-section of nanoparticles. Here, $\sigma_{abs}+\sigma_{sca}=\sigma_{ext}.$$\sigma_{sca}$ and $\sigma_{ext}$ stand the scattering and extinction cross-sections, respectively. Correspondingly, $Q_{abs}$, $Q_{sca}$ and $Q_{ext}$ stand the absorption efficiency, the scattering and extinction efficiencies, respectively. For spherical nanoparticles, $\sigma_{abs}=Q_{abs}\cdot \pi R^{2}$($\sigma_{sca}=Q_{sca}\cdot \pi R^{2},\sigma_{ext}=Q_{ext}\cdot \pi R^{2}$), which shows that $\sigma_{abs}$ is a quantity with dimensions of area [23].

The steady-state temperature distribution of the ith nanoparticle isolated in a homogeneous medium is considered. The details of the mechanism explanation and process are given in Reference [15]. For $\left|r-r_{i}\right|>R$,

(2) $$T\left(r\right)=\frac{Q_{i}}{4\pi \kappa_{s}\left|r-r_{i}\right|}+T_{\infty }.$$

For $\left|r-r_{i}\right|\leq R$,

(3) $$T\left(r\right)=\frac{Q_{i}}{4\pi \kappa_{s}R}+T_{\infty }.$$

where $\kappa_{s}$ stands the thermal conductivity of the surrounding medium, $T_{\infty }$ stands the temperature at which the distance is infinite (ambient temperature).

In the case of an array structure, the increased temperature at the position vector r in the outer space of the particles is provided by the collection of other j nanoparticles.

(4) $$\Delta T\left(r\right)=\sum_{j=1}^{N^{2}}G\left(r,r_{j}\right)Q_{j},$$

where

(5) $$G\left(r,r_{j}\right)=\frac{1}{4\pi \kappa_{s}\left|r-r_{j}\right|}.$$

The increased temperature inside the ith particle is

(6) $$\Delta T_{i}=\sum_{\begin{array}{l} j=1\\ j\neq i \end{array}}^{N^{2}}G\left(r_{i},r_{j}\right)Q_{j}+\frac{Q_{i}}{4\pi \kappa_{s}R}.$$

The first term of the above formula is the temperature contribution of $N^{2}-1$ particles around ith particle. The second term is self-contribution of ith particle. Therefore, Equation (6) can also be written as

(7) $$\Delta T_{i}=\Delta T_{i}^{ext}+\Delta T_{i}^{self}.$$

## 3. Results and Discussions

The optical properties of the Ag nanoparticle array were investigated carefully. In the following section, the influences of the radius, spacing and number of the particles and the illumination polarization angle on the absorption, near-field enhancement and photo-thermal temperature distribution are discussed in detail. The main parameters we set are as follows: The refractive index function file of Ag is given in Reference [24]. The refractive index of ambient medium is 1.33. The thermal conductivity of the medium is $0.599W/\left(m\cdot K\right)$ (considered in the water environment). The irradiance of incident light is $1.27\times 10^{8}W/m^{2}$.

### 3.1. Optical Properties of Array Structure

#### 3.1.1. The Influence of Particle Spacing

**Figure 2:**
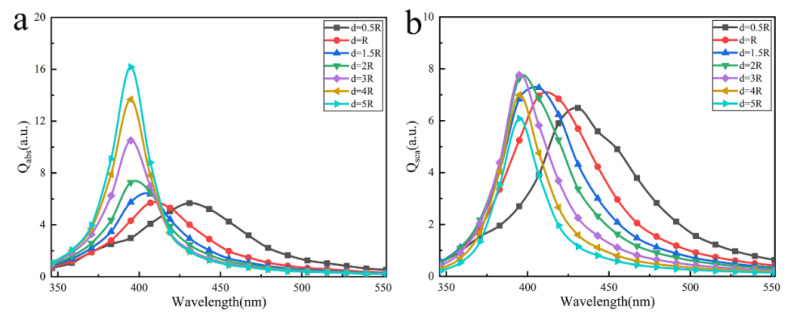
The absorption and scattering efficiencies of the 3×3 square array of Ag nanoparticles at different particle spacing with radius R=14nm : (**a**) absorption efficiency (**b**) scattering efficiency.

**Figure 3:**
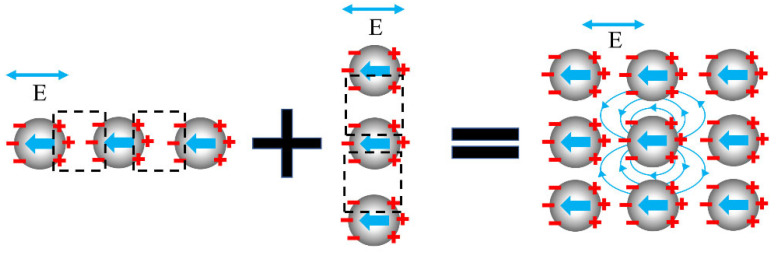
Schematic diagram of near-field coupling between particles in array under horizontal polarization.

In the case of y-polarized incident light, we calculated and analyzed the effects of particle spacing d on the optical properties of the array with 3×3 particles. Figure 2 shows the relationship of absorption and scattering efficiencies versus the particle spacing. The results show that the absorption and scattering peaks were broadened and redshifted with the decrease of the particle spacing, especially when d≤2R. It is considered that the surface plasmon phenomenon occurs when light is incident upon the array. In our case, each nanoparticle can be simply regarded as point dipoles, and the electron cloud inside them produces reciprocating oscillation behavior under the action of the electric field from incident light, as shown in Figure 3. While the nanoparticle spacing is close, the free electron oscillation inside each particle has different driven external field due to the scattering field from the surrounding particles, leading to the more oscillation frequencies of the free electronics. Multi-frequency of electron oscillation causes a broadened resonance peak. Considering the charge distribution of the particles, a decrease in the particle spacing weakens the restoring force of free electron oscillations inside particles due to the charge interaction of adjacent particles parallel to polarization (see Figure S1). Moreover, the resonance frequency decreases, and the resonance peak red-shifts. The weakening of the restoring force leads to the decrease in the amplitude of the electron oscillation. The decrease in the amplitude denotes the decrease in the absorption, and absorption efficiency becomes smaller. Generally speaking, the scattering energy includes the contribution of the first scattering from the particle and the multiple scattering between particles. Comparing the absorption efficiency, the intension of the scattering efficiency changes little when the particle spacing changes. The main reason is the scattering contribution of the light passing particle’s surface for the first time, not the multiple scattering between particles.

**Figure 4:**
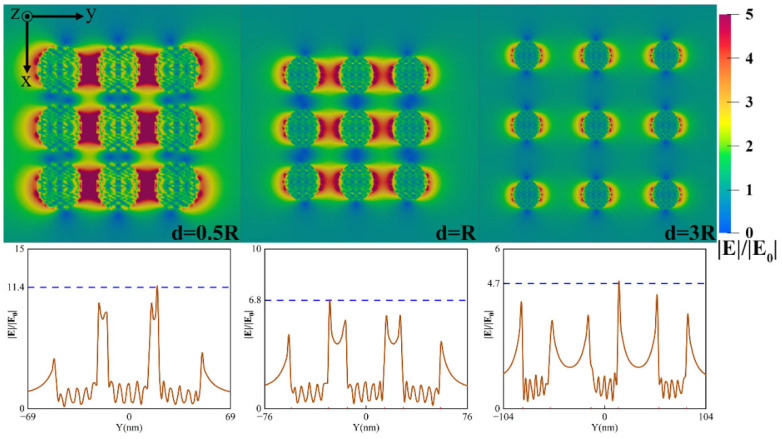
Near-field enhancement of the 3×3 square array of Ag nanoparticles with the radius of 14 nm and the incident light wavelength of 532nm: near-field distribution with different particle spacing (the top) and field enhancement factor curve along the y-axis of the array center (the bottom).

Figure 4 shows the near-field distribution at different particle spacings. The results show that the ‘hot spots’—strong electric field enhancement regions—appear in the particle gaps in the direction of polarization, especially when the particle spacing is smaller than the particle radius. ‘Hot spots’ become weaker gradually as the particle spacing increases, and electric field enhancement regions are limited to the light polarization direction near the surface of nanoparticles. ‘Hot spots’ exit because near-field coupling occurs between the nanoparticles when the particle spacing is small.

#### 3.1.2. The Influence of Particle Radius

**Figure 5:**
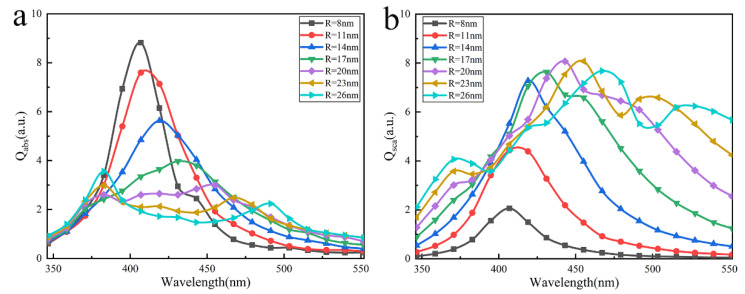
The absorption and scattering efficiencies of the 3×3 square array of Ag nanoparticles with different particle radiuses with spacing d=10nm : (**a**) absorption efficiency (**b**) scattering efficiency.

Similarly, in the case of y-polarized incident light, we calculated and analyzed the effects of particle radius R on the optical properties. Figure 5 shows the relationship of absorption and scattering efficiencies versus the particle radius. The results show that the absorption and scattering peaks are broadened and redshifted with the increase of the particle radius. Especially when the radius is 23 nm and 26 nm, two absorption peaks appear in Figure 5a and three scattering peaks appear in Figure 5b. While the nanoparticles’ radius increases, the driven electric field of incident light in the particle has a nonuniform phase and causes the phase difference of electron movement inside the particle, and ‘Phase delay’ effects occur. Moreover, each particle also has inconsistent oscillation frequencies due to the influence of the multiple scattering field. These lead to the broadening of the resonance peak. For the resonance peak shifting, considering the electric field force exerted by adjacent particles' charges (see Figure S2), the restoring force of free electron oscillations inside particles decreases as the number of surface free net charges of the neighboring particles increase. Therefore, the resonance frequency decreases and the resonance peak red-shifts as the particle radius increases. As we know, increasing particle radius leads to a decrease in absorption efficiency and an increase in scattering efficiency for a single particle (see Figure S3). The same occurred in nanoparticle arrays. On the one hand, the decrease in amplitude of the electron oscillation denotes the energy of electrons absorbing electromagnetic waves decreases. On the other hand, the contact area between particles and light increases with the particle's radius. Therefore, the absorption peak falls while the scattering peak rises when increasing the particle radius. For single particle, 2×2 and 3×3 arrays, we found that the peak number of scattering or absorption increases with particle number (see Figure 5 and Figure S3).

**Figure 6:**
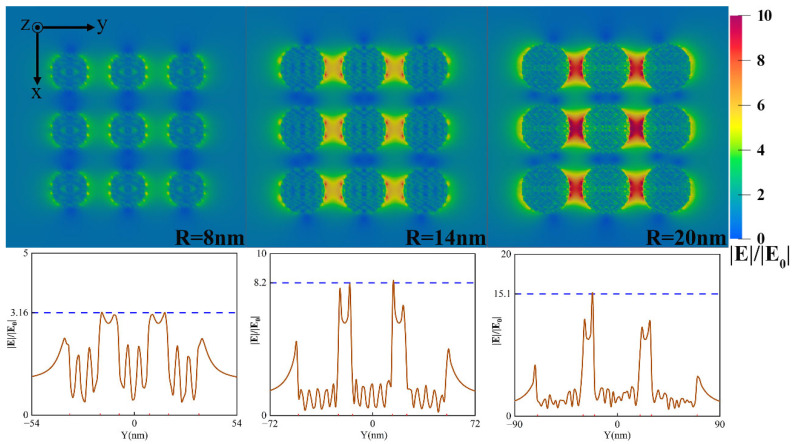
Near-field enhancement of the 3×3 square array of Ag nanoparticles with the particle spacing of 10nm and the incident light wavelength of 532 nm: near-field distribution with different radius (the top) and field enhancement factor curve along the y-axis of the array center (the bottom).

Figure 6 shows the near-field distribution when the particle radius varies. The result shows that ‘hot spots’ appear in the polarization direction of particle gaps with the increase in particle radius. Firstly, the absorption and scattering efficiencies of arrays with different radii are different at the wavelength of 532 nm, which affects the field or energy distribution around particles. Secondly, the number of free electrons in the particle goes up with the increase in particle radius, and more free electrons take apart in the oscillation due to drive by incident light, so the coupling between particles is significantly improved. However, different phenomena may occur at other wavelengths.

#### 3.1.3. The Influence of Illumination Polarization

**Figure 7:**
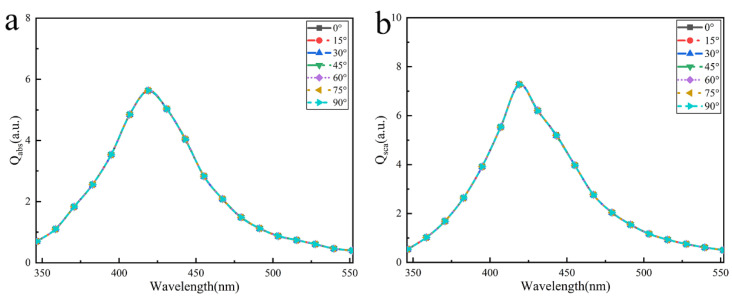
The absorption and scattering efficiencies of the 3×3 square array of Ag nanoparticles at different illumination polarization angles with particle radius R=14nm and particle spacing d=10nm : (**a**) absorption efficiency (**b**) scattering efficiency.

Furthermore, we investigated the influences of different illumination polarization on the optical properties of the array with 3×3 particles. Figure 7 shows the absorption and scattering efficiencies spectra of the Ag nanoparticle array. The result shows that the absorption and scattering efficiencies of the structure remain entirely unchanged as the polarization angle of the incident light increases from 0° to 90°. Firstly, the sphere is a highly centrosymmetric structure, and Ag nanoparticles in the array are isotropic and uniform materials. Therefore, the dependence of the polarization angle only needs to be considered as the particle arrangement position. Secondly, the square array conforms to orthogonal symmetry. When polarized light at any angle illuminates the array, polarization orthogonally decomposed into transverse and longitudinal modes [25]. To sum up the above two points, it is easy to conclude that the transverse and longitudinal modes of the absorption and scattering efficiencies are equal in the square array. Therefore, the absorption and scattering efficiencies are naturally independent of the incident polarization.

**Figure 8:**
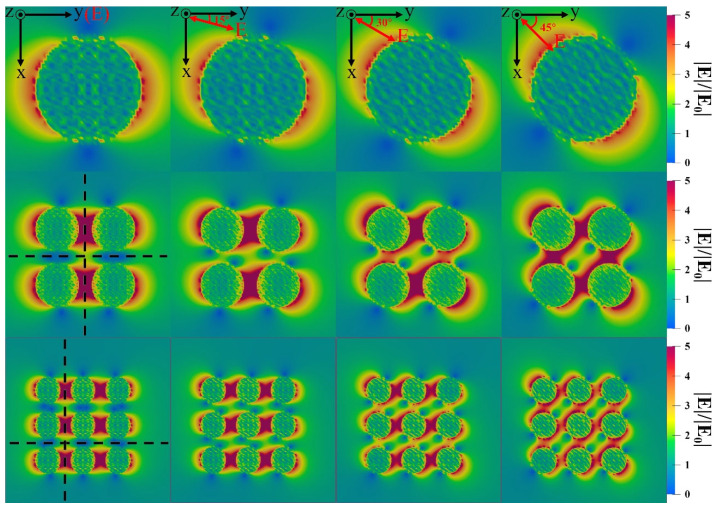
Near-field distribution of single and square arrays at different illumination polarization angles with the incident light wavelength of 532nm: the single (R=20nm), 2×2 array (R=20nm, d=10nm ) and 3×3 array (R=14nm, d=10nm ).

Then, we investigated the near-field enhancement effect at different illumination polarization angles from 0° to 45°. The near-field enhancement of the single Ag particle, 2×2 and 3×3 arrays of nanoparticles were calculated, shown in Figure 8. It is easy to observe that the direction of the near-field enhancement region rotates with the illumination polarization angle and the rotation is completely synchronous for a single nanoparticle. For arrays, it is noted that ‘hot spots’ tend to remain in the gap between nearest neighboring particles along the y-direction (or x-direction), although their intensity varies with the deflection of the polarization direction.

**Figure 9:**
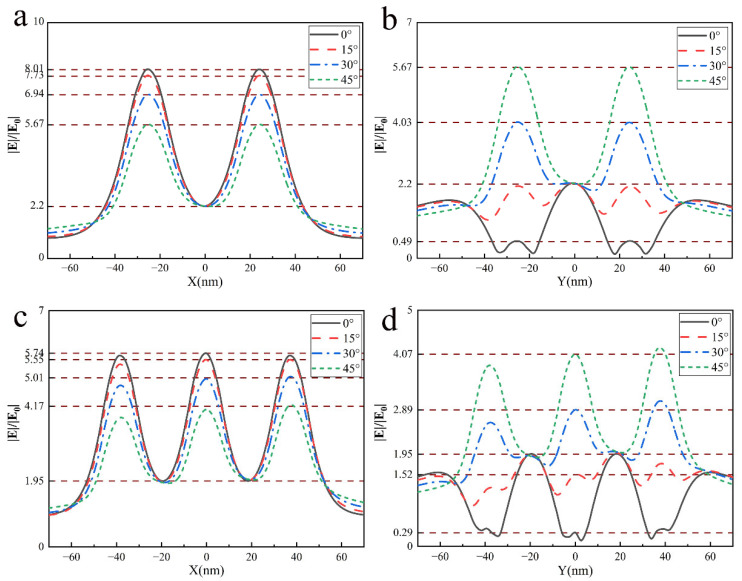
Enhancement factor curve at the dotted line in Figure 8 (**a**) x-direction of 2×2 array, (**b**) y-direction of 2×2 array (**c**) x-direction of 3×3 array, (**d**) y-direction of 3×3 array.

Interestingly, we found that the near-field intensity of the center of any four adjacent particles is kept constant when the illumination polarization changes. We call these points ‘constant spots’. Figure 9 shows the field enhancement factors along the dotted lines in Figure 8. We found that near-field enhancement remains invariable in ‘constant spots’ when the polarization changes, whether with 2×2 or 3×3 arrays. However, ‘hot spots’ arrive the strongest at the polarization direction of y-direction or x-direction, and monotonically decrease with the increase of the illumination polarization angle. The main reason is that the electron cloud oscillation inside the nanoparticles varies with illumination polarization. When the illumination polarization angle is located at the x-axis or y-axis, the distance of the surface oscillation charges between particles' surfaces arrives at a minimum, and the near-field enhancement reaches a maximum in the gap among particles due to the superposition of electric field excited by surface charges of neighbor particles. Interestingly, some researchers reported polarization independent of the photo-thermal temperature or absorption in nanostructures [25,26]. Here, we found polarization shielding or polarization independent of near-field enhancement in nanoparticle arrays. Our findings may contribute to the study of the near-field invariance of nano structures and provide new considerations for near-field modulation and control.

### 3.2. Photo-Induced Heating of Array Structure

In this section, the thermal Green's function method is used to investigate the photo-induced heating of spherical Ag nanoparticle arrays. To discuss the central temperature and temperature state of the array, ζ, a dimensionless parameter to indicate the state of temperature is defined as [11]

(8) $$\zeta =\frac{\Delta T_{0}^{self}}{\Delta T_{0}^{ext}},$$

where $\Delta T_{0}^{self}$ stands the temperature contribution of the central particle itself, and $\Delta T_{0}^{ext}$ stands the temperature contribution of the surrounding $N^{2}-1$ nanoparticles. If ζ≫1, indicates that self-contribution is dominant, and the structure is a temperature confinement regime. If ζ≪1, it indicates that external contribution is dominant, and the structure is a temperature delocalization regime. When multiple scattering is negligible, the external field $E_{i}^{ext}$ is equal to the illuminating incident field $E_{i}^{inc}$. Based on the reference [11], we draw the expression without considering the multiple scattering effect between particles as

(9) $$\zeta =\frac{d+2R}{3(N-1)R}.$$

**Figure 10:**
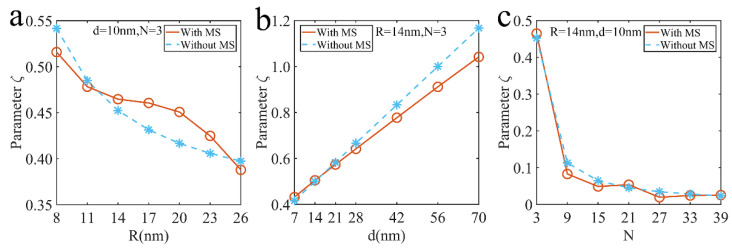
The relationship between the dimensionless parameter ζ and the parameter of the array structures: (**a**) radius, (**b**) particle spacing, (**c**) particle number.

We used the thermal Green’s function method to calculate the dimensionless parameter under multiple scattering and compared with Equation (9) (see Figure 10) and explored this dimensionless parameter of the array varying with the radius, particle spacing and particle number. From the calculation results in Figure 10, it can be seen that the state parameters with multiple scattering are between 0.02 and 1.04, which indicates that the structure under consideration is basically in the temperature delocalization state. It is obvious that the dimensionless parameter decreases with the increase of radius and particle number, shown in Figure 10a,c. Interestingly, the parameters increase linearly with particle spacing.

**Figure 11:**
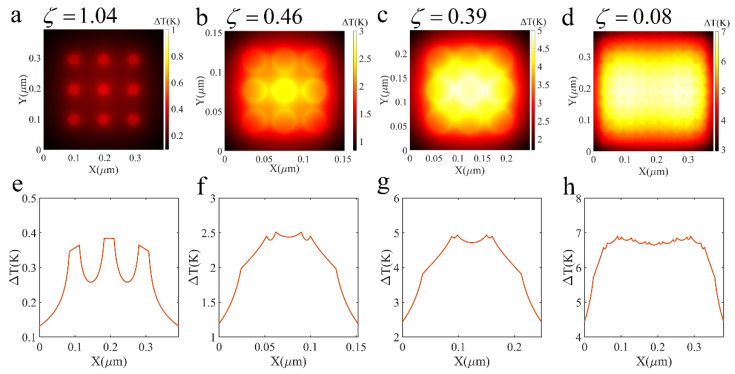
Photo-induced heating of Ag nanoparticle array illuminated by uniform 532nm light with light irradiance $I=1.27\times 10^{8}W/m^{2}$: (**a**) 3×3 array (R=14nm, d=70nm, $Q_{abs}=0.2635$ ), (**b**) 3×3 array (R=14nm, d=10nm, $Q_{abs}=0.5601$ ), (**c**) 3×3 array (R=26nm, d=10nm, $Q_{abs}=1.0154$ ), (**d**) 9×9 array (R=14nm, d=10nm, $Q_{abs}=0.5601$ ), (**e**–**h**) Temperature variation curve of horizontal center line.

In order to observe the temperature distribution of the array more intuitively, we calculated the temperature distribution of some structures and the temperature variation curve of the horizontal center line, shown in Figure 11. Each particle can be regarded as an individual nano heat source when ζ=1.04. However, as ζ decreases, the thermal coupling effect becomes obvious, and the temperature of the array tends to be more uniform. So, we can draw the conclusion that the parameter ζ of our structures denotes the temperature delocalization regime.

**Figure 12:**
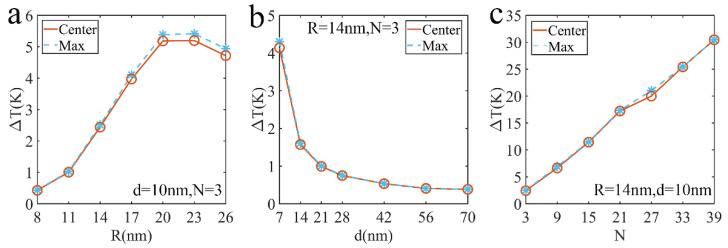
The central and maximum temperature of Ag nanoparticle array structure: (**a**) radius dependent, (**b**) particle spacing dependent, (**c**) particle number dependent.

Similarly, to analyze the temperature distribution in detail, we explored the center and maximum temperature of the array varying with the radius, particle spacing and particle number. We found that the maximum temperature is not the position of the array center, which is reflected in Figure 11. As for maximum and center temperatures, they significantly rise quickly as the particle radius increases when 8nm≤R≤20nm, shown in Figure 12a. However, the effect of plasmonic coupling leads to the decrease in temperature when R>20nm. The center and maximum temperature of the array decrease when the particle spacing increases, and it increases linearly with the number of particles, as shown in Figure 12b,c.

**Figure 13:**
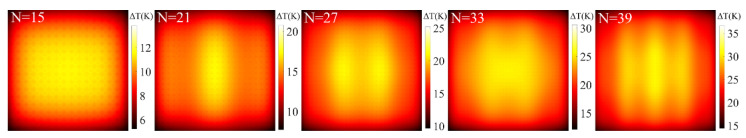
Temperature distribution under the effect of plasmonic coupling.

It is always a major subject to control the temperature distribution by using nanoscale particles, and it has been reported that specific temperature distribution can be achieved by designing structures [27]. This can be used to manipulate single cell adhesion [28] and other fields [29]. It has broad application prospects. In the study, we found that, as shown in Figure 13, the plasma coupling effect led to an unconventional temperature distribution in the array, which varied with the number of particles. Therefore, we consider that adjusting the plasma coupling mode can also be a way to achieve temperature distribution regulation.

## 4. Conclusions

The near-field enhancement and photo-thermal temperature distribution of Ag nanoparticle arrays were investigated by using DDA and thermal Green's function methods. The results show that in the polarization direction, ‘hot spots’ appear in the particle gap when the particle spacing is smaller than the particle radius. The particle radius also affects the generation of ‘hot spots’. It is noted that the absorption and scattering efficiencies of the array are unchanged when the illumination polarization angle is changed, and the position of the ‘hot spots’ do not rotate with the polarization direction of the illumination light. Moreover, we find that ‘constant spots’, which are located at the array center composed of four adjacent particles, and the factor of near-field enhancement stay constant in these spots when the illumination polarization varies. As for photo-induced heating, we determined a parameter ζ to describe the temperature delocalization state. Each particle can be regarded as an individual nano-heat source when the dimensionless parameter ζ=1.04. However, as ζ decreases, the thermal coupling effect becomes obvious, and the temperature of the array tends to be more uniform. Interestingly, the maximum temperature is not always located at the position of the array center. However, the center and maximum temperature vary with the parameter of the array with a similar law. Here, we propose that the coupling effect of a scattered light field between particles may also be used to regulate temperature distribution. This work provides some theoretical considerations for the field manipulation of nanostructures.

## Data Availability

Not applicable.

## Supplementary Materials

The following supporting information can be downloaded at: https://www.mdpi.com/article/10.3390/nano12213924/s1, Figure S1: The absorption and scattering efficiencies of chain composed by 3 Ag nanoparticles with different particle spacing and radius. (a,b) The chain is perpendicular to polarization. (c,d) The chain is parallel to polarization; Figure S2: The absorption and scattering efficiencies of chain composed by 3 Ag nanoparticles with different particle radius and spacing d = 10 nm (a,b) The chain is perpendicular to polarization. (c,d) The chain is parallel to polarization; Figure S3: The absorption and scattering efficiencies of the different structure of Ag nanoparticle with different particle radius (a,b) a single Ag nanoparticle (c,d) 2 × 2 square array of Ag nanoparticles with particle spacing *d* = 10 nm.
