# Supplementary material for: Optical Properties of Ag Nanoparticle Arrays: Near-Field Enhancement and Photo-Thermal Temperature Distribution
Source: Nanomaterials (Basel). 2022 Nov 7;12(21):3924. doi: 10.3390/nano12213924 (PMC9656548; doi:10.3390/nano12213924)
Supplement: Supplementary file 1 [file nanomaterials-12-03924-s001.zip › nanomaterials-1957552-supplementary.pdf]

Supplementary Materials

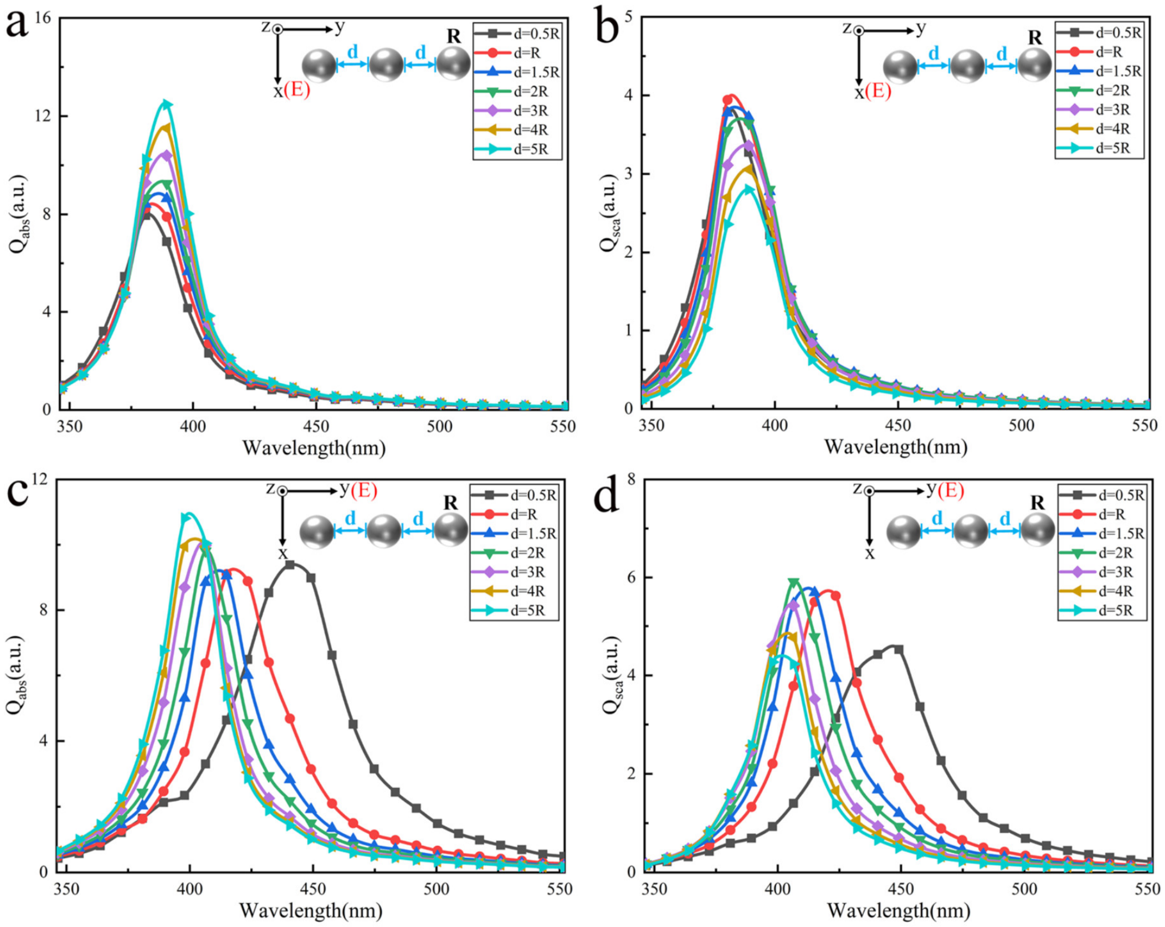

Figure S1. The absorption and scattering efficiencies of chain composed by 3 Ag nanoparticles with different particle spacing and radius. (a, b) The chain is perpendicular to polarization. (c, d) The chain is parallel to polarization.

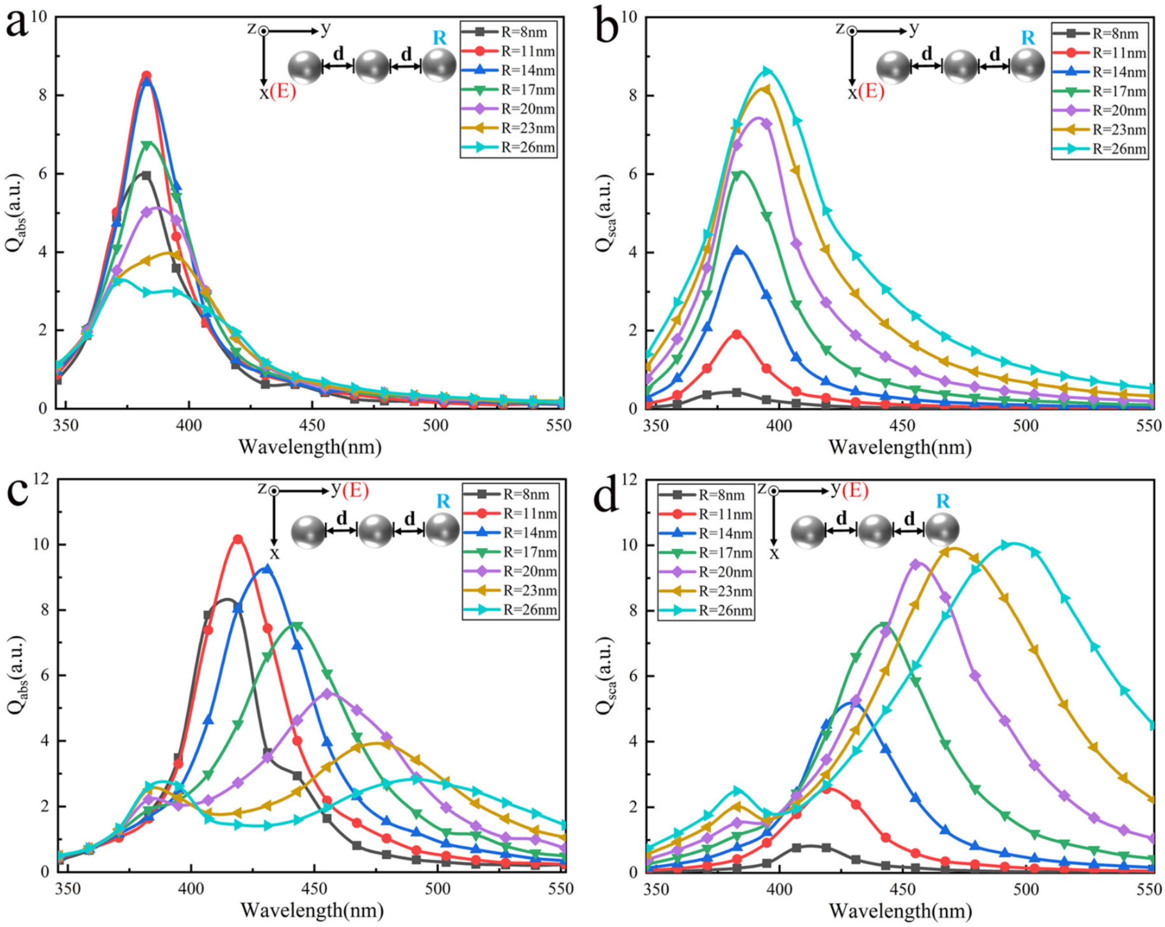

Figure S2. The absorption and scattering efficiencies of chain composed by 3 Ag nanoparticles with different particle radius and spacing *d*=10 nm (a, b) The chain is perpendicular to polarization. (c, d) The chain is parallel to polarization.

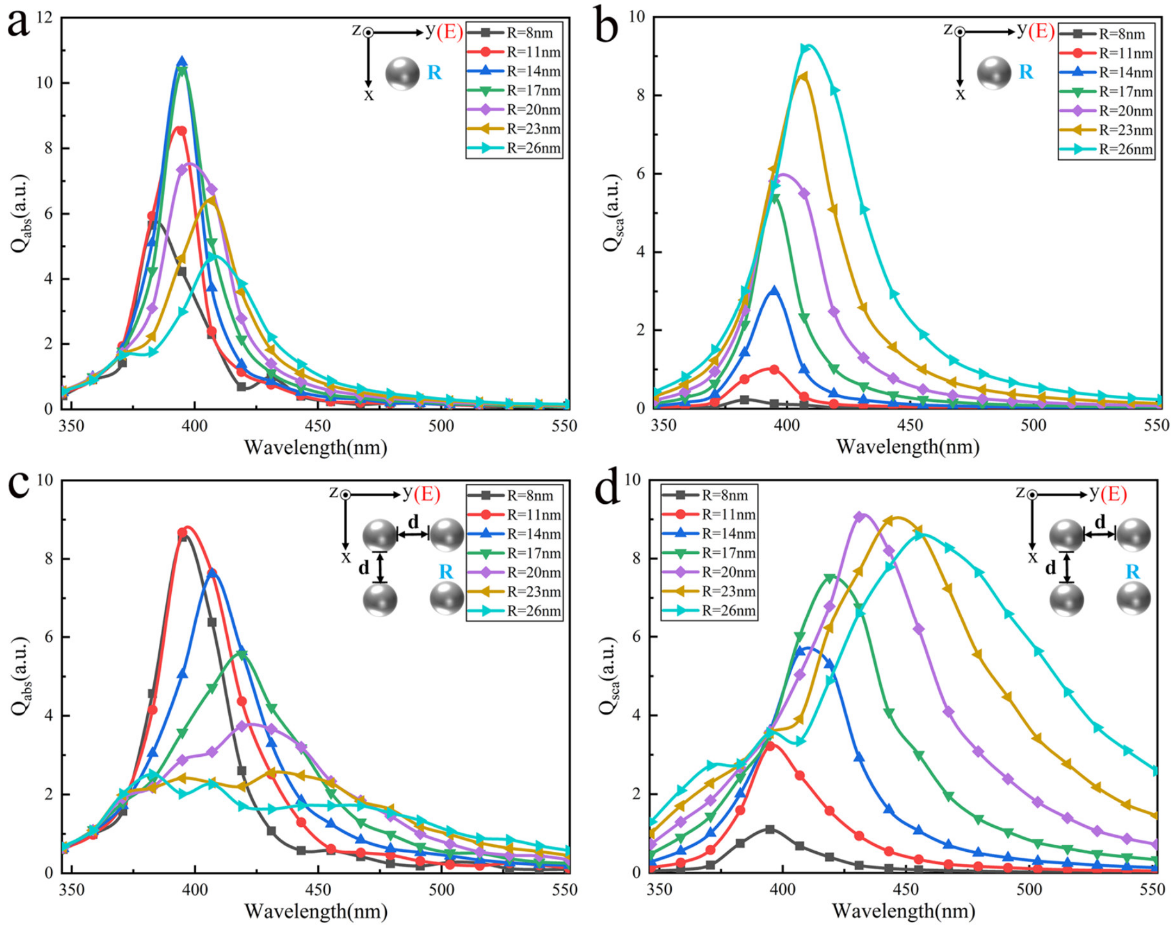

Figure S3. The absorption and scattering efficiencies of the different structure of Ag nanoparticle with different particle radius (a, b) a single Ag nanoparticle (c, d) 2×2 square array of Ag nanoparticles with particle spacing *d*=10 nm.
